# Effector/memory CD4 T cells making either Th1 or Th2 cytokines commonly co-express T-bet and GATA-3

**DOI:** 10.1371/journal.pone.0185932

**Published:** 2017-10-31

**Authors:** Arundhoti Das, Vidya Ranganathan, Danish Umar, Shipra Thukral, Anna George, Satyajit Rath, Vineeta Bal

**Affiliations:** National Institute of Immunology, New Delhi, India; Mie University Graduate School of Medicine, JAPAN

## Abstract

Naïve CD4 T (NCD4T) cells post-activation undergo programming for inducible production of cytokines leading to generation of memory cells with various functions. Based on cytokine based polarization of NCD4T cells in vitro, programming for either ‘Th1’ (interferon-gamma [IFNg]) or ‘Th2’ (interleukin [IL]-4/5/13) cytokines is thought to occur via mutually exclusive expression and functioning of T-bet or GATA-3 transcription factors (TFs). However, we show that a high proportion of mouse and human memory-phenotype CD4 T (MCD4T) cells generated in vivo which expressed either Th1 or Th2 cytokines commonly co-expressed T-bet and GATA-3. While T-bet levels did not differ between IFNg-expressing and IL-4/5/13-expressing MCD4T cells, GATA-3 levels were higher in the latter. These observations were also confirmed in MCD4T cells from FVB/NJ or aged C57BL/6 or IFNg-deficient mice. While MCD4T cells from these strains showed greater Th2 commitment than those from young C57BL/6 mice, pattern of co-expression of TF was similar. Effector T cells generated in vivo following immunization also showed TF co-expression in Th1 or Th2 cytokine producing cells. We speculated that the difference in TF expression pattern of MCD4T cells generated in vivo and those generated in cytokine polarized cultures in vitro could be due to relative absence of polarizing conditions during activation in vivo. We tested this by NCD4T cell activation in non-polarizing conditions in vitro. Anti-CD3 and anti-CD28-mediated priming of polyclonal NCD4T cells in vitro without polarizing milieu generated cells that expressed either IFNg or IL-4/5/13 but not both, yet both IFNg- and IL-4/5/13-expressing cells showed upregulation of both TFs. We also tested monoclonal T cell populations activated in non-polarizing conditions. TCR-transgenic NCD4T cells primed in vitro by cognate peptide in non-polarizing conditions which expressed either IFNg or IL-4/5/13 also showed a high proportion of cells co-expressing TFs, and their cytokine commitment varied depending on genetic background or priming conditions, without altering pattern of TF co-expression. Thus, the model of mutually antagonistic differentiation programs driven by mutually exclusively expressed T-bet or GATA-3 does not completely explain natural CD4 T cell priming outcomes.

## Introduction

Peripheral naïve CD4 T cells are exposed over the animal’s lifetime to a variety of immunogens in a variety of micro-environmental contexts, leading to accumulation of a secondary/memory CD4 T cell pool diverse in both antigenic specificities and effector potential. During priming and differentiation, memory CD4 T cells are thought to commit to various alternative programs that enable them to make different effector cytokines upon restimulation. These include the so-called ‘Th1’ (IFNg), ‘Th2’ (IL-4-, IL-5, IL-13), ‘Th17’ (IL-17, IL-22), ‘Th9’ (IL-9) or the induced regulatory T (iTreg) programs. Cytokines [1–8], co-stimulatory and accessory molecules [9–11] as well as antigen-presenting cell (APC) types [12–14] make a significant contribution to the memory fate determination of NCD4T cells post-activation. In contrast, transcription factor (TF) Mina, a member of the jumonji C (JmjC) protein family described to confer Th2 bias [15], functions in T cell intrinsic fashion for cell fate determination post-activation.

The mechanistic insights in the regulation and control of CD4 T cell memory fate determination programs [2,3,16] have commonly come from work in controlled conditions in vitro in which uniform microenvironments bring about relatively homogeneous differentiation and generation of memory cells [10–12]. This approach using polarizing conditions has shown that early production of IFNg or IL-4 during priming, either from APCs or from responding naive cells [17,18], leads to the induction of T-bet or GATA-3 respectively, and that these signaling pathways suppress each other, so that T-bet-expressing cells do not express GATA-3 and vice versa, leading to expression of either ‘Th1’ or ‘Th2’ cytokines from the primed cells [19–21]. Such polar-differentiated memory cells are resistant to de-differentiation and plasticity [19]. However, there are many examples of the existence of plasticity, particularly in memory cells differentiated in vivo [1,20–23]. Following infection or vaccination, individual memory cells appear to show binary choices of cytokine programs [24,25], in that they make either, say, ‘Th1’ cytokines or ‘Th2’ cytokines and only uncommonly both [23].

As memory cells accumulate in vivo as an outcome of extremely complex and heterogeneous priming conditions we were interested in asking the following question. Are the binary choices of ‘Th1’ versus ‘Th2’ cytokine programs made in memory cells primed in vivo, driven by the mutually exclusive expression of T-bet versus GATA-3? We address this by showing that in vivo generated memory CD4 cells from mice and humans or antigen-specific effector CD4 T cells generated following immunization and capable of producing either IFNg or IL-4/5/13 but not both co-express T-bet and GATA-3 in high proportion of cells. In fact, such exclusively IFNg-producing or IL-4/5/13-producing memory cells show only subtle quantitative differences in their relative levels of T-bet and GATA-3. Surprisingly, this situation is mimicked by cell-free priming of naive cells in vitro with anti-CD3 and anti-CD28 under non-polarizing conditions; even when such primed cells make either IFNg or IL-4/5/13, they co-express T-bet and GATA-3 with subtle quantitative differences in their relative levels. Nonetheless, the genetic or environment-mediated differences reported in the relative prominence of IFNg versus IL-4/5/13 seen in memory cells generated in vivo are maintained in such in vitro primed cells, and appear to be independent of cytokines and TCR repertoires. These data suggest that the model of mutually antagonistic Th1/Th2 differentiation programs driven by mutually exclusively expressed T-bet or GATA-3 does not completely explain natural CD4 T cell priming outcomes, where subtle quantitative differences in relative levels of T-bet and GATA-3 may lead to binary Th1/Th2 commitment with efficiencies modulated by T cell-intrinsic factors.

## Materials and methods

### Mice

All mice used were obtained from the Jackson Laboratory and bred in the Small Animal Facility of the National Institute of Immunology. Mice in this facility are periodically tested for specific pathogens and were found to be negative. Mice of either sex aged between 4 to 8 weeks of age, except aged mice which were at least 18 months of age, were used for all the experiments. The mouse strained used were C57BL/6 (B6), B6.SJL, aged B6, IFNg-/- and BALB.b (all H-2b strains), FVB/NJ (FVB, H-2q), IL-4-GFP (4Get, H-2d) and BALB/c (H-2d). OT-II (H-2b), OT-II mice bred onto BALB.b background for ten generations (H-2b) and DO11.10 (H-2d) mice that have transgenic TCR restricted to H-2Ab or H-2Ad and specific for an ovalbumin (Ova) peptide (amino acids 323–339 (Ova-II), Invitrogen, USA) were also used. Mice were euthanized by cervical dislocation in all the experiments. For each set of experiment between 3–6 mice were used per group.

### Source of human peripheral blood mononuclear cells (PBMCs)

PBMCs were separated by Ficoll-Hypaque (Ficoll-PaqueTM PREMIUM, GE Healthcare) density gradient centrifugation either from venous blood collected from healthy young adult volunteers after consent, or from buffy coat residua after platelet separation of blood from anonymized healthy adult blood bank donors (age range 22–45 years) of All India Institute of Medical Sciences, New Delhi.

### Reagents

RPMI 1640 medium (Biological Industries) containing 10% FCS, antibiotics, sodium pyruvate and non-essential amino acids (all reagents from Sigma Aldrich) was used for all cultures. Various fluorochrome or biotin coupled antibodies to detect mouse CD4, CD25, CD44, CD45.1, CD45.2, CD62L, DO11.10-TCR clonotype (KJ1-26), IFNg, IL-4, IL-5, IL-13, T-bet, GATA-3, TCRValpha2, TCRVbeta5 and human CD4, CD45RO, CCR7, CD25, IFNg, IL-13, T-bet, GATA-3 along with appropriate isotype controls (BD Biosciences, eBioscience, BioLegend, Cell Signaling Technology) were used. Secreted IL-4, IL-13 and IFNg from mouse and human cell cultures were detected using ready enzyme-linked immunosorbent assays (eBiosciences). Cultures were supplemented with recombinant human IL-2 (Roche) where necessary. Dual color ELISpot kits (R&D Systems) were used to detect IFNg and IL-4 producing mouse CD4 cells. Brefeldin, Phorbol Myristate Acetate (PMA) and ionomycin were procured from Sigma. For human and mouse T cell activation in vitro culture grade anti-CD3 and anti-CD28 (eBiosciences) were used. For polarizing cultures recombinant cytokines IL-4, IFNg, IL-12 and antibodies anti-mouse IL-4, anti-mouse IFNg and anti-mouse IL-12 (all from eBiosciences) were used.

### Flow cytometric purification of naive and memory cells

Mouse spleen CD4 T cells were sorted as naive (CD4+CD44-CD62L+CD25-) to include only conventional T cells and exclude Treg cells or memory (CD4+CD44+). Depending on the requirement, spleen cells from 2–3 age- and sex-matched mice were pooled and cells from a single sort were used for a given experiment and such independent experiments were repeated. Human CD4 T cells were sorted from PBMCs of independent donors as naive (CD4+CD45RO-CD25-CCR7+) or memory (CD4+CD45RO+). Cells were rested for 2–3 h at 37 C before use in various experiments. Sorting was done under sterile conditions (BD FACSAria III). Spleen cells from 4Get mice identified as CD4+CD44+ were sorted into green fluorescent protein (GFP)+ and GFP- populations for some assays. Normally, purity was ≥ 95%.

### Priming and/or restimulation of CD4 T cells in vitro

Naive CD4 T cells from various strains of mice, as specified for each experiment, were activated without any polarizing milieu, by optimal concentrations of plate-coated anti-CD3 and anti-CD28 (3 μg/ml each) for 72 h. Cells were then harvested, rested in IL-2 (5 U/ml) for 24 h, and used for subsequent assays. The in vitro priming protocol for naive T cells was optimised with many titrations of days for priming and for resting as well as IL-2 concentrations. IL-2 concentrations were titrated to maximise cell survival and live cell yields without influencing Th1/Th2 cytokine balance. Rest period titration was done to achieve return of cells to baseline in terms of cytokine secretion, so that stimulation-induced production could be unambiguously followed. For detection of intracellular cytokines and TFs, cells were treated with phorbol myristate acetate (50 ng/ml) and Ionomycin (500 ng/ml) (P+I) for 5 h and Brefeldin A (5 ng/ml) for the last 2.5 h before permeabilisation and staining with anti-cytokine and anti-TF antibodies. For analyzing secreted cytokines, cells were restimulated with anti-CD3 and anti-CD28 for 24 h, and cytokines were assayed in culture supernatants. Human naive cells were similarly primed, except that higher IL-2 concentrations (50 U/ml) were used.

For priming in vitro under Th1- or Th2- polarizing conditions, mouse naïve CD4 cells were activated with plate-coated anti-CD3 and anti-CD28 as above for 72 h in presence of recombinant murine IFNg (10 ng/ml), recombinant murine IL-12 (10 ng/ml) and anti-mouse IL-4 (10 μg/ml) for Th1 polarization, or recombinant murine IL-4 (50 ng/ml), anti-mouse IFNg (10 μg/ml) and anti-mouse IL-12 (10 μg/ml) for Th2 polarization.

Sorted memory cells, both mouse and human, were activated in vitro with plate-coated anti-CD3 and anti-CD28 (3 μg/ml each) for 24 h for supernatant cytokine assays, or with PMA and ionomycin with addition of Brefeldin A as above for detection of intracellular cytokines and TFs.

For priming TCR-transgenic naive cells, Ova-II peptide and syngeneic bone-marrow dendritic cells (BMDCs) prepared as described previously [26] were used instead of anti-CD3 and anti-CD28 as above, both for priming and for restimulation.

For immunization of mice with Ova-II peptide, sorted naive CD4 T cells from DO11.10 mice, 0.3 million per mouse, were transferred i.v. to BALB/c mice. Twenty four hours later recipient mice were either immunized with ovalbumin (50 μg/mouse in the footpad) on alum or left without immunization. Seven to 9 day later mice were euthanized and popliteal and inguinal lymph nodes were harvested. Cells were stimulated with 3 μg/ml of Ova-II peptide for 24 h. In the last 5 hours PMA and Ionomycin were added and intracellular staining for cytokines and TFs was done as described above.

For ELISpot assays, memory cells generated in vivo and sorted, or naive cells primed for 72 h and then rested for 24 h in IL-2, were restimulated with irradiated splenocytes (1:4 ratio of T cells:splenocytes) and soluble anti-CD3 (100 ng/ml), or with Ova-II peptide (3 μg/ml) for TCR-transgenic CD4 T cells, for 24 h in ELISpot plates.

### ELISA and ELISpot assays for cytokines

Secreted cytokines were detected by commercially available cytokine detections kits (eBiosciences) as mentioned above, essentially following recommended protocols. A reference standard run in parallel was used to calculate the cytokine concentrations.

IFNg, IL-4 and dual cytokine (IFNg & IL-4) producers were identified using the mouse IFNg/IL-4 dual color ELISpot kit from R&D Systems. T cells and APCs were added to pre-coated ELISPOT plates in ratios as above and stimulated as appropriate for 24 h. Detection antibodies and substrates were added as recommended by the manufacturer. Colored spots were read using an ELISpot reader (Autoimmun Diagnostika GmbH) using the dual color analysis settings. If no dual positive spots were recorded data are shown only for IFNg and IL-4.

### Flowcytometric staining and data analysis

Cells were stained with fluorochorme labelled antibodies added in optimum concentrations for 30 minutes on ice. For intracellular staining surface stained cells were permeabilised and optimum concentrations of antibodies for cytokines and transcription factors added. Appropriate irrelevant antibodies coupled to fluorophores were used as controls in each experiment. Between NCD4T and MCD4T cells there was no difference in staining profile of isotype control antibodies for GATA-3, T-bet, IFNg or IL-4/5/13 staining as illustrated by staining profiles from one experiment (S1A Fig). Additional profiles for isotype controls for human naive and memory cells and in vitro primed mouse NCD4T cells are also included in supplementary figures. In some figures, data normalized to isotype controls is shown but in most cases, because of near identical staining with isotype controls MFI values were directly used for calculations. Data were analyzed by FlowJo software (Treestar, USA).

### RT-qPCR assays for tbx21 and gata3

RNA was extracted from ~5 million naive, in vitro primed for 72 h or sorted MCD4 cells activated for 24 h using Trizol (Invitrogen) following recommended procedures. In brief, after RNA extraction the purity was determined by Nanodrop 2000 spectrophotometer (Thermo). Only RNA samples with 260/280 ratio of 1.9–2.1 and a 260/230 ratio >2 were used for analysis. All samples were DNase treated. Using 1 μg RNA, cDNA synthesis was done by reverse transcription kit (Promega). The *tbx21* and *gata-*3 primers were designed in-house, custom-synthesized (Sigma) and validated before use for RT-qPCR. Ribosomal protein *l7* was used as a housekeeping gene [27–31]:

*l7* forward primer: AGC TCA TCT ATG AGA AGG C
*l7* reverse primer: AAG ACG AAG GAG CTG CAG AAC
*tbx21* forward primer: AGA ACT TTG AGT CCA TGT ACG
*tbx21* reverse primer: TAA CTG TGT TCC CGA GGT G
*gata3* forward primer: ACC GGG TTC GGA TGT AAG
*gata3* reverse primer: GAC AGT TCG CGC GCA GGA TGT

RT-qPCR was performed in triplicate each time (Eppendorf Mastercycler ep realplex 4). The optimized conditions were: 94°C for 1 min (denaturation), 60°C for 30 sec (annealing), 72°C for 45 s (elongation) for a total of 40 cycles, followed at the end by 72°C for 5 mins (extension). Ct values obtained for *tbx21* (T-bet) and *gata3* (GATA-3) were normalized with L7 values and **ΔΔ**Ct values were calculated [32] and plotted to show enhancement in signal above NCD4T cell values. Naive cells served as controls for sorted memory cells and in vitro primed T cells. Each experiment consisted of RNA extracted from paired samples. NCD4T and MCD4T cells sorted on a given day formed a pair. Alternatively, from sorted NCD4 cells one aliquot was immediately stored in Trizol while the other aliquot was stored at the end of activation. Multiple independent repeats of such experiments provided Ct values.

### Ethical permissions

All mouse experiments were carried out after approval of the Institutional Animal Ethics Committee. All human experiments were carried out after approval of the Institutional Human Ethics Committee. Informed written consent was obtained from all the donors before withdrawal of the blood from ante-cubital vein.

### Statistical methodology

For statistical analysis, Student’s ‘t’ test was used, either in unpaired format when comparing different mouse groups, or in paired format when comparing naïve versus memory phenotype CD4 T cell properties. Values of p <0.05 were considered statistically significant.

## Results

### Memory CD4 T cells generated in vivo producing either IFNg or IL-4/5/13 but not both, co-express T-bet and GATA-3

**Fig 1:**
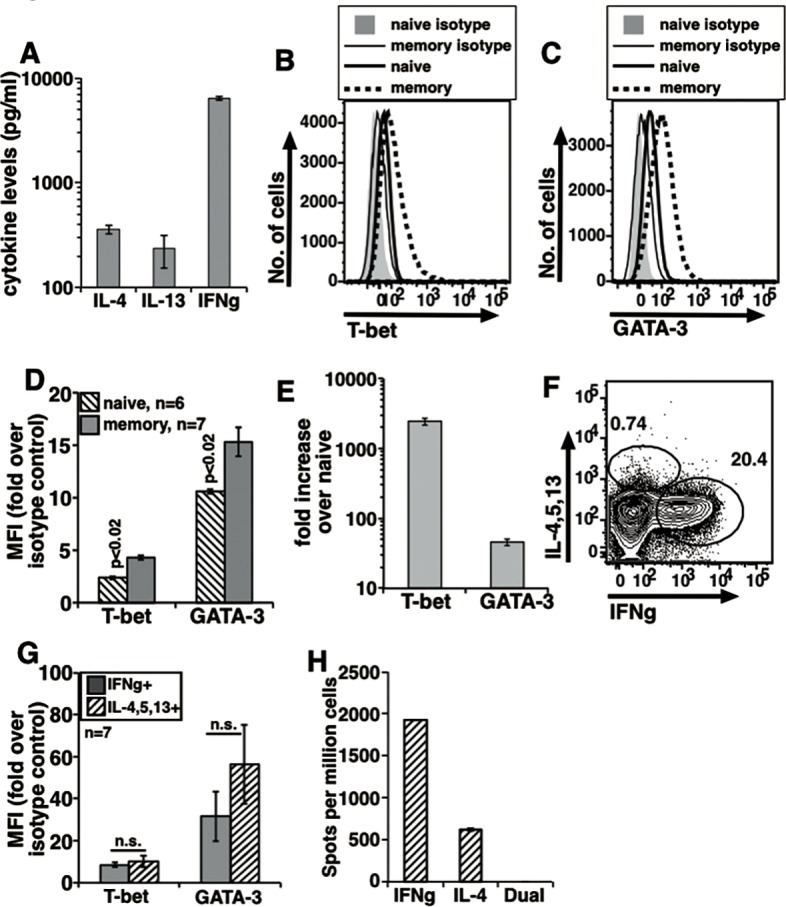
In vivo generated memory cells from B6 mice producing either IFNg or IL-4/5/13 co-express T-bet and GATA-3. (A) Data from 3 independent experiments to show secreted cytokines in response to 0.1 μg/ml anti-CD3 (mean ± s.e.) with constant dose of anti-CD28. Values for naive cells <100 pg/ml. Representative histograms to show T-bet (B) and GATA-3 (C) levels in sort-purified naive and memory cells after P+I treatment in vitro. (D) Data from independent experiments showing fold increase in MFI values over isotype controls as indicated (mean ± s.e.). (E) Data from 5 independent experiments compiled to show increase in relative mRNA levels above NCD4T cells from B6 mice at 24 h. (F) A representative plot to show intracellular expression of IFNg and IL-4/5/13 in sorted MCD4T cells treated with P+I in vitro. (G) Data from independent experiments compiled to show relative T-bet and GATA-3 MFI increases over isotype controls in IFNg+ and IL-4/5/13+ cells as from (F) (mean ± s.e.). (H) Dual color ELISpot data from one experiment showing number of spots (mean ± s.e.). Pattern representative of 3 independent experiments.

We examined the levels of T-bet and GATA-3 expression and the expression potential for the major cytokines they regulate, namely, IFNg versus IL-4/IL-13, in naturally generated MCD4T cells in vivo by stimulating them in vitro. NCD4T (CD4+CD44-CD25-CD62L+) and MCD4T (CD4+CD44+) cells were sorted from B6 splenic tissue. When these cells were stimulated with plate-coated anti-CD3+anti-CD28 for 24 h and culture supernatants analyzed for secreted cytokines, memory cells showed the presence of various levels of IFNg, IL-4 and IL-13 as expected (Fig 1A). Cytokine levels from NCD4T cells similarly activated were below 100 pg/ml.

Both T-bet and GATA-3 levels were higher in MCD4T cells as compared to NCD4T cells, both at the protein level by flow cytometry (Fig 1B–1D) and at the mRNA level by RT-qPCR assays (Fig 1E). The pattern of expression of both T-bet and GATA-3 was unimodal (Fig 1B and 1C). Two-color plots for T-bet and GATA-3 in NCD4T and MCD4T cells showed that unimodal expression of T-bet and GATA-3 produced a co-expression pattern in MCD4T cells (S1B Fig). When MCD4T cells were stained for intracellular cytokines after P+I activation, a large proportion expressed IFNg while a small population expressed IL-4/5/13 (Fig 1F). There were very few, if any, MCD4 cells which produced both the cytokines. We then compared T-bet and GATA-3 levels in the MCD4T cells expressing either IFNg or IL-4/5/13. Notably, both T-bet and GATA-3 expression patterns in IFNg-producing and IL-4/5/13-producing MCD4T cells were also unimodal leading to their co-expression (S1C Fig). Quantitative analysis of the relative protein levels of T-bet and GATA-3 in IFNg-expressing versus IL-4/5/13-expressing MCD4T cells from multiple experiments showed that neither T-bet nor GATA-3 levels differed between them significantly (Fig 1G). Near absence of naturally generated MCD4T cells producing both IFNg and IL-4 was confirmed in dual-color ELISpot assays (Fig 1H).

Together, these data showed that a large majority of memory cells generated in vivo in B6 mice co-express T-bet and GATA-3, yet do not co-express IFNg and IL-4/5/13. Even in MCD4T cells expressing IFNg or IL-4/5/13, T-bet levels did not differ, while GATA-3 levels differed quantitatively though not reaching statistical significance.

### Co-expression of T-bet and GATA-3 is also commonly observed in memory T cells from other mouse strains

**Fig 2:**
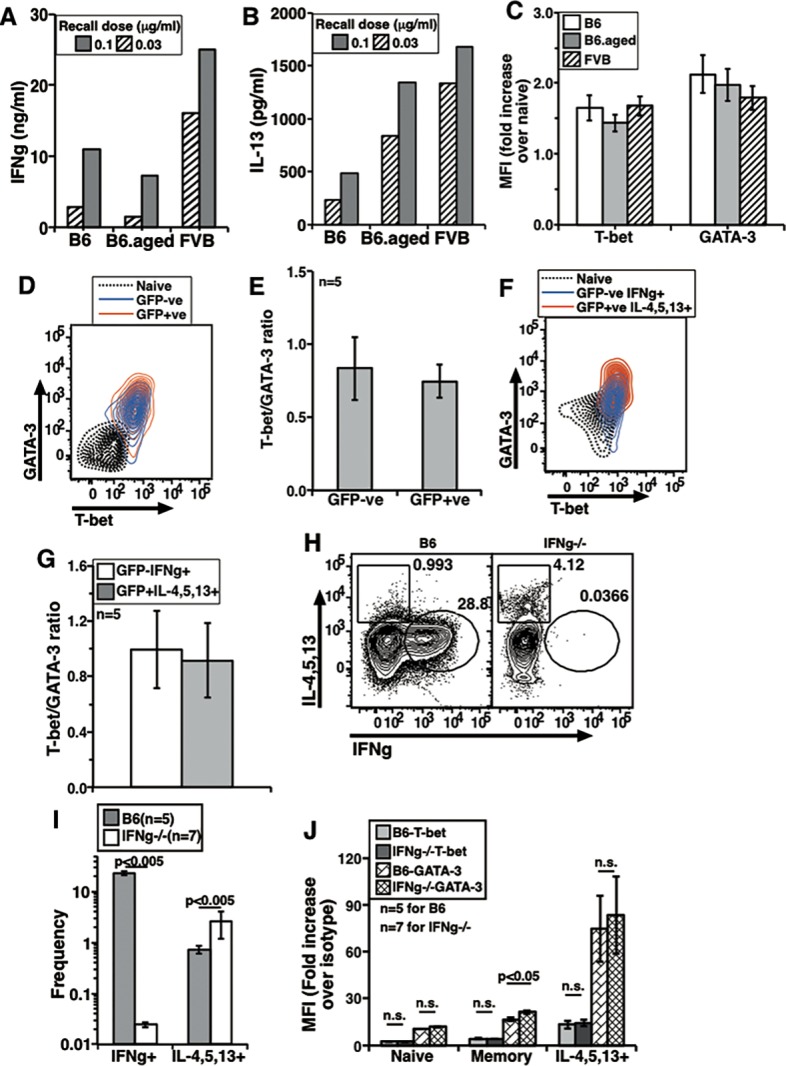
In vivo generated memory cells isolated during steady state from multiple strains show co-expression of T-bet and GATA-3. Dose response curve for IFNg (A) and IL-13 (B) production by sorted MCD4T cells stimulated for 24 h in vitro from groups as shown. One representative experiment out of three with similar trends shown. (C) Data from independent experiments to show relative T-bet and GATA-3 MFI increases over naive cells in B6, B6.aged and FVB memory cells (mean ± s.e., n = 3). The isotype control profiles showed no difference in naive and memory cell staining in each strain. (D) A two color plot to show T-bet and GATA-3 expression in NCD4T, GFP-ve MCD4T and GFP+ve MCD4T cells from 4Get mice. (E) T-bet/GATA-3 MFI ratios from GFP-ve MCD4T and GFP+ve MCD4T cells obtained from independent experiments (mean ± s.e.). (F) A representative profile of NCD4T, GFP-ve IFNg+ve and GFP+ve IL-4/5/13+ve cells from 4Get mice for T-bet and GATA-3 expression. (G) T-bet/GATA-3 MFI ratios from GFP-ve IFNg+ve MCD4T and GFP+ve IL-4/5/13+ve MCD4T cells obtained from independent experiments (mean ± s.e.). (H) Intracellular cytokine staining of MCD4T cells from B6 and IFNg-/- mice stimulated with P+I. (I) Data from independent experiments showing frequencies of IFNg+ve and IL-4/5/13+ve cells from P+I stimulated MCD4T cells from B6 and IFNg-null mice (mean ± s.e.). (J) Data from independent experiments to show relative T-bet and GATA-3 MFI increases over isotype controls in NCD4T, MCD4T and IL-4/5/13+ve MCD4T from B6 and IFNg-/- mice (mean ± s.e.).

We next tested MCD4T cells for levels and potential co-expression of T-bet and GATA-3 in situations known to vary in the IFNg/IL-4 balances generated in CD4 T cell responses. Aged mice are reported to generate a relative prominence of Th2 cytokines compared to young animals [33,34]. The independent inbred FVB/NJ strain of mice is also reported to generate more Th2-cytokine-dominated CD4 T cell responses [35,36]. We therefore purified and analyzed splenic NCD4T and MCD4T cells from young B6, B6.aged (~18 months), and FVB/NJ mice. When these cells were stimulated with plate-coated anti-CD3+anti-CD28 for 24 h and culture supernatants analyzed for secreted cytokines, MCD4 cells from all three strains showed a dose dependent increase in IFNg production as seen in a representative experiment (Fig 2A). Aged B6 and FVB/NJ MCD4T cells showed greater prominence of IL-13 compared to memory cells from young B6 mice, as expected (Fig 2B). Cytokine levels from similarly activated NCD4T cells were below 100 pg/ml. Dual-color ELISpot assays provided additional support showing the relative prominence of IL-4 in memory cells on recall from aged B6 and in FVB/NJ mice (S1D Fig), but in all strains, memory cells produced either IFNg or IL-4 on recall, with no detectable co-producers. Both T-bet and GATA-3 showed unimodal expression pattern in MCD4T cells from all three groups and two-color plots for naive and memory cells from all the strains showed co-expression despite the difference in cytokine pattern observed (S1E Fig). Data compiled from multiple independent experiments and normalized for respective NCD4T cell values showed that relative increases in T-bet and GATA-3 levels in the MCD4T cells from all 3 strains were similar to each other despite differences in the cytokine production pattern (Fig 2C).

Since the intracellular staining frequencies for IL-4/5/13 were generally low, we also tested 4Get, a mouse strain where cells activated to express IL-4 co-express GFP [37]. In comparison with non-transgenic wild-type BALB/c mice, splenic CD4+CD44+ MCD4T cells from 4Get mice specifically showed a clearly detectable GFP-expressing sub-population (S2A Fig). From pooled splenic cell suspensions, we sorted NCD4T and MCD4T cells from BALB/c mice and NCD4T, GFP-ve MCD4T and GFP+ve MCD4T cells from 4Get mice. A representative profile of GFP+ve and GFP-ve MCD4 cells from 4Get mouse spleen cells showed non-overlapping curves for the sorted populations (S2B Fig). In the absence of P+I stimulation the expression of both IFNg and IL-4/5/13 were negligible (S2C Fig). GFP-ve and GFP+ve MCD4T cells from 4Get mice showed unimodal distribution and co-expression of T-bet and GATA-3 (Fig 2D). T-bet/GATA-3 ratios of GFP-ve and GFP+ve MCD4 cells following P+I activation were similar to each other as seen from compiled data from independent experiments (Fig 2E). P+I activation also lead to expression of cytokines in both GFP-ve and GFP+ve populations. However, GFP-ve IFNg+ve and GFP+ve IL-4/5/13+ve populations showed unimodal expression of T-bet and GATA-3. Overlays of these two populations with NCD4T cells from 4Get mice showed co-expression of TFs (Fig 2F). When T-bet/GATA-3 rations of GFP-ve IFNg+ve and GFP+ve IL-4/5/13+ve populations were calculated from multiple independent experiments, there was no difference (Fig 2G). Thus, these data further confirmed that MCD4T cells expressing either ‘Th1’ or ‘Th2’ cytokines did not show exclusive expression of T-bet and GATA-3 respectively.

IFNg is expressed at low but significant levels early during naive cell activation [38,39], and has been reported to be a major regulator of the Th1/Th2 cytokine commitment balance in responding naive cells via its ability to induce T-bet expression [3]. We therefore tested cytokine commitment and T-bet/GATA-3 expression in memory cells from B6 versus IFNg-null mice. When MCD4T cells were stimulated with plate-coated anti-CD3+anti-CD28 for 24 h and secreted cytokines measured in culture supernatants, memory cells from IFNg-null mice showed greater prominence of IL-4 and IL-13 compared to memory cells from WT mice, as expected (S2D Fig). When sorted memory cells were stimulated with P+I and stained for cytokines, MCD4T cells from B6 mice showed high frequency of IFNg+ cells whereas those from IFNg-null mice did not show IFNg (Fig 2H). However MCD4T cells from IFNg-null mice showed higher frequency of IL-4/5/13+ve cells as compared to those from B6 mice (Fig 2H). The frequencies of IL-4/5/13+ cells as well as IFNg+ cells in MCD4T cells from B6 and IFNg-null mice in independent experiments remained different and statistically significant (Fig 2I). Again, despite this difference in cytokine commitment, memory cells from IFNg-null mice showed unimodal patterns of T-bet and GATA-3 expression in two-color plots indistinguishable from the expression patterns seen in memory cells of B6 mice (S2E Fig). Further, extent of upregulation of T-bet and GATA-3 was examined in naive, memory and IL-4/5/13+ve memory cells following P+I activation in independent experiments. Data show that relative GATA-3 levels were higher in memory population from IFNg-null mice as compared to B6 mice, however, T-bet levels were comparable (Fig 2J). Thus, IFNg modulated cytokine commitment in memory cells without drastically changing T-bet or GATA-3 levels.

### Human memory cells show co-expression of T-bet and GATA-3

**Fig 3:**
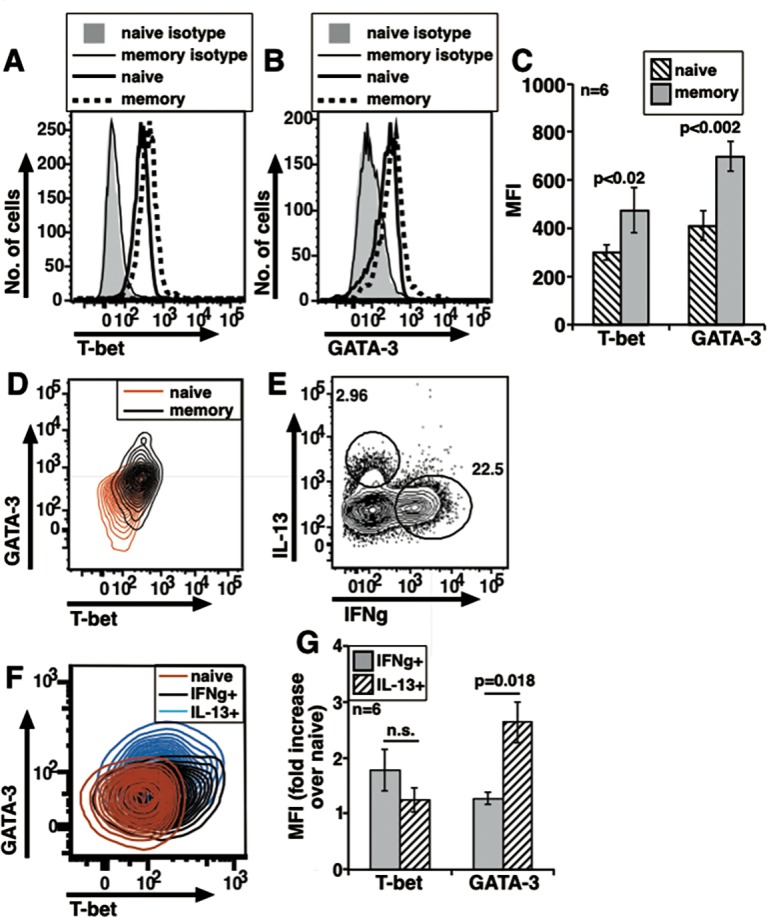
In vivo generated memory cells from human blood which express either IFNg or IL-13 co-express T-bet and GATA-3. (A) Representative histograms to show T-bet levels in sorted naive and memory cells. (B) Representative histograms to show GATA-3 levels in sorted naive and memory cells. (C) Data from independent donors compiled to show MFI values as indicated (mean ± s.e.). (D) Two-color plot representative of one donor to show T-bet and GATA-3 levels in naive and memory cells. (E) A representative plot to show intracellular expression of IFNg and IL-13 in P+I treated cells. (F) Two color plot representing one donor to show T-bet and GATA-3 levels in naive, IFNg+ memory and IL-13+ memory cells. (G) Data from individual donors compiled to show relative T-bet and GATA-3 MFI increases over naive cells in IFNg+ and IL-13+ cells (mean ± s.e.).

We extended these findings to human memory cells from adult volunteer donors. NCD4T (CD4+CD45RO-CD25-CCR7+) and MCD4T (CD4+CR45RO+) cells were purified from donor PBMCs. Like mouse MCD4T cells, human MCD4T cells showed higher levels with unimodal distribution of T-bet and GATA-3 expression than NCD4T cells did and the differences were significant (Fig 3A–3C). Similar to the staining pattern for isotype controls for mouse cells, isotype controls for human naive and memory cells for T-bet and GATA-3 did not differ significantly from each other (S3A and S3B Fig). Two color plot with overlay of naive and memory cells showed that unimodally distributed T-bet and GATA-3 expressing memory cells showed co-expression of TFs (Fig 3D), similar to mouse memory cells. When memory cells were stained for intracellular cytokines after P+I activation, a large proportion showed presence of IFNg while a small population expressed IL-13, but hardly any cells could be detected expressing both cytokines (Fig 3E). The isotype control stainings for IFNg and IL-13 were also comparable between naive and memory cells (S3C and S3D Fig). Thus, a large majority of human memory cells generated in vivo which express either IFNg or IL-13 but not both nonetheless co-express T-bet and GATA-3. Interestingly, while IFNg+ and IL-13+ human MCD4T cells also showed unimodal expression of the TFs indicative of co-expression (Fig 3F), relative GATA-3 levels were higher in IL-13-expressing human MCD4T cells whereas T-bet levels were no different between IFNg-expressing and IL-13-expressing MCD4T cells (Fig 3G). These data are inconsistent with a model of necessarily mutually antagonistic expression of T-bet and GATA-3 for binary Th1 versus Th2 cytokine commitment.

### Effector T cells generated following immunization show co-expression of T-bet and GATA-3 in Th1 or Th2 cytokine producing cells

**Fig 4:**
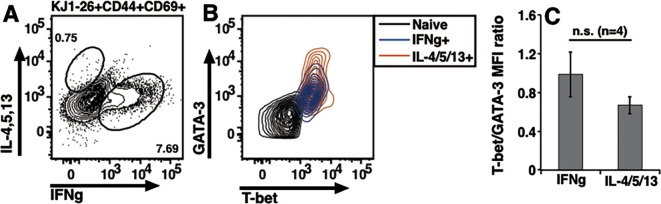
Ova-II-specific effector/memory DO11.10 cells expressing IFNg or IL-4/5/13 co-express T-bet and GATA-3. (A) A representative plot from draining lymph node cells to show IFNg and IL-4/5/13 producing cells on recall. Lymph node cells gated as KJ1-26+CD69+CD44+ were analyzed for intracellular cytokine staining. (B) IFNg+ or IL-4/5/13+ cells from (A) showing expression of T-bet and GATA-3 as compared to naive CD4 T cells. (C) Ratios of T-bet and GATA-3 MFIs were calculated for IFNg+ or IL-4/5/13+ cells. (Mean ± s.e.).

In addition to analyzing steady state memory T cells in vivo we examined antigen-specific T cell response following immunization. Recall response of draining lymph node cells from BALB/c mice which had received DO11.10 cells and were immunized with ovalbumin were analyzed. KJ1-26-expressing cells were identified and gated as DO11.10 cells. In response to Ova-II peptide recall in vitro CD69+CD44+ DO11.10 cells were gated. A subset of these showed presence of IFNg or IL-4/5/13 (Fig 4A). When these subsets were analyzed for their T-bet and GATA-3 expression the curves were overlapping and the levels were much higher than the naive cells (Fig 4B). When T-bet/GATA-3 ratios of MFIs were calculated they were not different from each other (Fig 4C) indicating that Th1 or Th2 cytokine producing antigen-specific effector T cell population generated in vivo co-expresses T-bet and GATA-3.

### Effector T cells generated in vitro and expressing either IFNg or IL-13 but not both nonetheless co-express T-bet and GATA-3

We next tested if co-expression of T-bet and GATA-3, and yet not of IFNg and IL-13, could also be observed when NCD4T cells were primed in vitro to generate activated effector population. Previously published work contributing to the model of mutually antagonistic expression and function of T-bet and GATA-3 have made extensive use of naive CD4 T cell priming in vitro. Naive CD4 (CD4+CD44-CD25-CD62L+) T cells from B6 spleens were sort-purified and activated with plate-coated anti-CD3 and anti-CD28 for 72 h without any ‘polarizing’ conditions. These primed cells, the majority of which had proliferated and upregulated CD44 expression (S3E and S3F Fig), were rested for 24 h in IL-2 (5 U/ml) before being tested for cytokine commitment. Addition of optimal amount of IL-2 during the rest period helped in better yields of viable cells but did not make any difference to the extent of upregulation of T-bet and GATA-3 in BALB/c naive CD4 cells activated in presence of anti-CD3 and anti-CD28 or DO11.10 TCR Tg cells activated by Ova-II peptide and BMDCs (S3G and S3H Fig).

**Fig 5:**
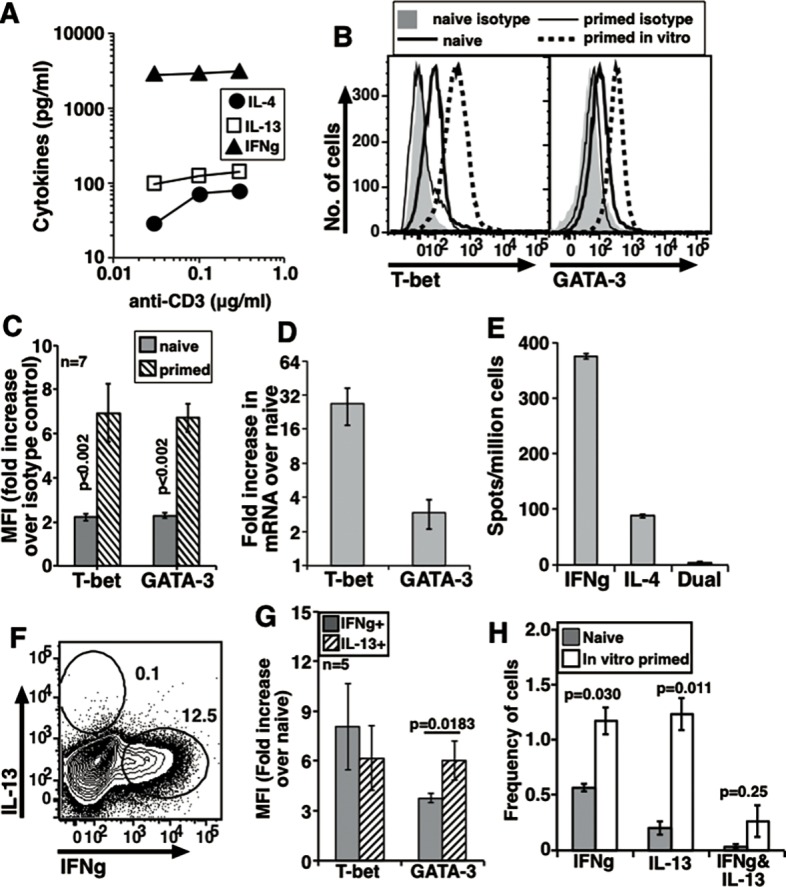
Naive CD4 T cells activated in non-polarizing conditions in vitro show pattern similar to memory cells generated in vivo. (A) Dose response curve for primed and recalled NCD4 T cells from B6 mice from one experiment to show secreted cytokine levels in primed and recalled cells. Data representative of >5 independent experiments. Cytokine values for naive cells were <10 pg/ml. (B) A representative profile to show T-bet and GATA-3 levels in naive and in vitro primed CD4 cells from B6 mice. (C) Data from 7 independent experiments compiled to show relative MFI values (fold increase over isotype control) for T-bet and GATA-3 (mean ± s.e.) from B6 mice. (D) Data from 5 sets of independent experiments compiled to show relative mRNA levels, normalized to NCD4T cell values, 72 h after priming (mean ± s.e.). (E) Dual color ELISpot assay to show number of spots (mean ± s.e.). Data representative of 4 independent experiments on B6 T cells. (F) Primed NCD4T cells from B6 mice show large presence of IFNg producing cells after P+I treatment. (G) Data from independent experiments on NCD4T cells from B6 mice compiled to show relative T-bet and GATA-3 MFI increases over naive cells in IFNg+ and IL-13+ cells (mean ± s.e.). (H) NCD4 T cells from BALB/c mice primed for 72 h in vitro and recalled to score single and dual cytokine producing cells. Frequency of cytokine producing cells from naive and primed T cell groups (mean ± s.e., n = 3).

When these in vitro-primed cells from B6 mice were stimulated with plate-coated anti-CD3 and anti-CD28 for 24 h, they secreted IFNg, IL-4 and IL-13 (Fig 5A), while naive cells similarly activated produced cytokine levels below 10 pg/ml.

As with in vivo generated MCD4T cells, both T-bet and GATA-3 levels were higher in cells primed in vitro versus unprimed NCD4T cells from B6 mice by flow cytometry (Fig 5B and 5C). There was only marginal, if at all, difference in MFI values for isotype controls for these cells (S3I and S3J Fig). At 72 h post-activation relative mRNA levels by real-time RT-PCR assays were much higher over signal in naive cells from B6 mice (Fig 5D). These in vitro-primed CD4 T cells which show unimodal upregulation of T-bet and GATA-3 show a pattern of co-expression in two color plots (S3K Fig). When these primed cells from B6 mice were tested for cytokine production by dual-color ELISpot assays, most cytokine-secreting cells produced either IFNg or IL-4, while very few could be scored as producing both (Fig 5E). When these in vitro-primed cells from B6 mice were P+I-activated and stained for intracellular cytokines, a large proportion showed presence of IFNg while a small population expressed IL-13, with no dual cytokine-expressing cells (Fig 5F). These signals were clearly detectable over isotype control staining (S3L and S3M Fig). Nonetheless, in vitro-primed cells expressing IFNg or IL-13 showed similar levels of T-bet, and while both groups expressed GATA-3, the levels were higher in the IL-13-expressing cells (Fig 5G). It should be noted that we did not detect any T cells producing both IFNg and IL-4/5/13, even when we examined NCD4T cells from BALB/c mice activated in vitro in similar conditions, where the frequency of IL-13 producing cells was relatively higher (Fig 5H).

Thus, even when primed CD4 T cells generated in vitro without any polarizing conditions differentiated into cells that produced either IFNg or IL-13 but not both, nonetheless co-expressed both T-bet and GATA-3, mimicking MCD4T cells generated in vivo.

### Effector cells generated in vitro under polarizing conditions do not co-express T-bet and GATA-3

We also confirmed that, in our hands, co-expression of T-bet and GATA-3 was not seen when naive CD4 T cells were primed in vitro in polarizing conditions, as observed in previous data contributing to the model of mutually antagonistic expression and function of T-bet and GATA-3. Naive cells were sort-purified and primed as above, but in the presence of either anti-IL-4, recombinant IFNg and recombinant IL-12 for ‘Th1’ differentiation, or of anti-IFNg, anti-IL-12 and recombinant IL-4 for ‘Th2’ differentiation as previously reported [40], or without any ‘polarizing’ conditions. To maintain the parity with non-polarizing activation protocol used, priming was restricted to 72 h. These primed cells were rested for 24 h in IL-2 (5 U/ml) before being tested for cytokine commitment.

**Fig 6:**
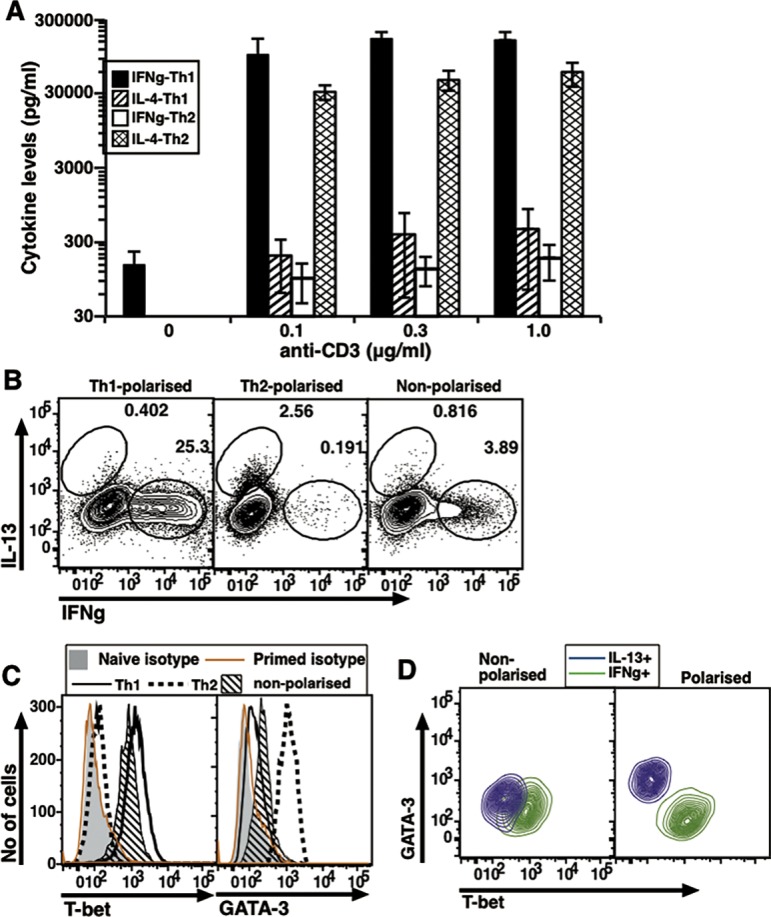
Polarized in vitro activation of naive CD4 cells shows no co-expression, but non-polarized does, of T-bet and GATA-3. (A) Cytokine production by in vitro Th1 and Th2 polarized cells from B6 mice. Data pooled from 3 experiments (mean ± s.e.). (B) A representative plot to show intracellular cytokine staining of polarized and non-polarized cells activated for 72 h. Data representative of 4 experiments. (C) Representative histograms to depict T-bet and GATA-3 levels in 72 h activated Th1 and Th2 polarized cells in comparison to non-polarized cells. Data representative of 3 experiments. (D) Representative two color plots to show T-bet and GATA-3 in IFNg+ and IL-13+ populations from panel B as overlays.

When in vitro-primed cells were re-stimulated with plate-coated anti-CD28 and titrating amounts of anti-CD3 for 24 h to examine secreted cytokines, ‘Th1’-polarized cells showed high IFNg production in a dose-dependent fashion whereas very low levels of IL-4 (Fig 6A). In contrast, Th2 polarized cells showed negligible IFNg production but increasing levels of IL-4 production in a dose-dependent fashion (Fig 6A).These cells were stimulated with P+I and examined for intracellular cytokines and TFs. After 72 hours of activation, ‘Th1’-polarized cells showed a sizeable proportion of cells expressing IFNg and a very small frequency of cells with IL-13 expression (Fig 6B). Th2-polarized cells showed hardly any IFNg-expressing cells but relatively higher proportion of IL-13 producing cells and non-polarized cells showed lower frequencies of IFNg-expressing and IL-13-expressing cells, with no cells co-expressing both IFNg and IL-13 (Fig 6B). Interestingly, IFNg levels per cell appeared higher in IFNg+ cells primed in ‘Th1’-polarizing conditions than in those primed in non-polarizing conditions, while IL-13 levels per cell showed no such distinction (S4A Fig).

With regard to T-bet and GATA-3 expression, while ‘Th1’-polarized cells had very high levels of T-bet and very low levels of GATA-3 and vice versa for ‘Th2’-polarized cells as expected, non-polarized cells showed relatively lower levels of both TFs as noted above (Fig 6C). Two-color plots of IL-13-expressing and IFNg-expressing cells from appropriately polarized cultures showed the expected either/or pattern of T-bet and GATA-3 expression, while IL-13-expressing and IFNg-expressing cells primed under non-polarizing conditions expressed both T-bet and GATA-3 at low levels (Fig 6D).

### Co-expression of T-bet and GATA-3 is commonly observed in CD4 cells primed in vitro

**Fig 7:**
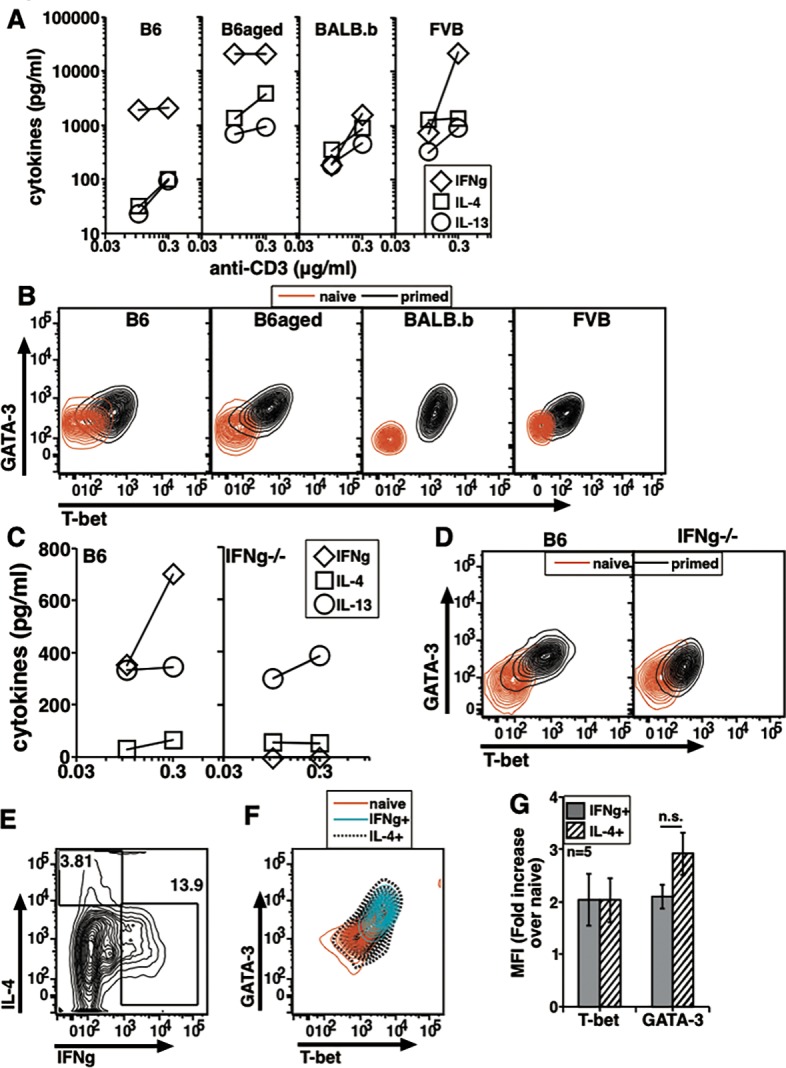
Cells activated in non-polarizing conditions in vitro from other mouse strains and humans show co-expression of T-bet and GATA-3. (A) Dose response curve from one experiment to show secreted cytokine levels in primed and recalled cells from B6, B6aged, BALB.b and FVB. Data representative of 3 independent experiments. (B) Representative two-color plots of T-bet and GATA-3 expression in naive and primed cells as overlays. Data representative of 5 experiments. (C) Dose response curve from one experiment to show secreted cytokine levels in primed and recalled cells from B6 and IFNg-/- mice. Data representative of 3 independent experiments. (D) A representative two-color plot of T-bet and GATA-3 expression in naive and primed cells from B6 and IFNg-/- mice as overlays. Data representative of 3 experiments. (E) A representative plot to show intracellular cytokine staining in P+I treated 72 h primed human NCD4T cells. (F) Two color plots of in vitro primed human CD4 cells to show T-bet and GATA-3 expression in indicated populations as overlays. (G) Data from independent donors compiled to show relative T-bet and GATA-3 MFI increases over naive cells in IFNg+ and IL-4+ human CD4 T cells.

We extended these findings to in-vitro priming of naive cells from other mouse strains. Naive cells from young and aged B6 mice, BALB.b and FVB/NJ mice were purified and primed in vitro as above. When these cells were re-stimulated with plate-coated anti-CD3+anti-CD28 for 24 h, cells from B6.aged mice, BALB.b mice and FVB/NJ mice produced higher levels of IL-4 and IL-13 than cells from young B6 mice did (Fig 7A), as observed with memory cells generated in vivo (Fig 2A and 2B). Dual-color ELISpot assays suggested that similar to in vitro primed NCD4T cells from B6 mice in vitro (Fig 5E) individual NCD4T cells from B6.aged, FVB and BALB.b mice expressed either IFNg or IL-4 on recall, not both (S4B Fig). Despite this difference in cytokine commitment, a large proportion of in vitro-primed cells from all strains showed unimodal increase in T-bet and GATA-3 expression levels which showed co-expression patterns in two-color plots (Fig 7B).

We also tested cytokine commitment and T-bet, GATA-3 expression in naive cells from B6 versus IFNg-null mice primed in vitro. When such in vitro-primed cells were stimulated with plate-coated anti-CD3+anti-CD28 for 24 h and secreted cytokines measured in culture supernatants, IL-4 and IL-13 levels showed no differences despite the absence of IFNg (Fig 7C), notably unlike memory cells generated in vivo (S2D Fig). Also, despite the absence of IFNg expression, in vitro-primed cells from IFNg-null mice showed unimodal upregulation and co-expression of T-bet and GATA-3 in two-color plots similar to that seen in in vitro-primed cells from B6 mice (Fig 7D).

Similarly, naive human CD4 T cells (CD4+CD45RO-CD25-CCR7+) sort-purified from human PBMCs, were activated in vitro for 72 h using anti-CD3+anti-CD28. When such in vitro-primed human CD4 cells were activated with P+I, some of them expressed IFNg and some IL-4, but none expressed both cytokines (Fig 7E). Yet, a large proportion of IFNg-expressing and IL-4-expressing cells showed overlapping expression patterns for T-bet and GATA-3 (Fig 7F). While relative T-bet levels were not different between IFNg-producing and IL-4-producing cells, GATA-3 levels were marginally higher but statistically insignificant in IL-4-producing cells (Fig 7G).

### Antigen-specific activation of TCR-transgenic naïve CD4 cells leads to co-expression of T-bet and GATA-3 but not of ‘Th1’ and ‘Th2’ cytokines

Since the in vitro priming protocol used so far, namely, plate-coated anti-CD3 and anti-CD28, is not physiological, we next examined if a more physiological naïve CD4 cell priming protocol in vitro could still result in co-expression of T-bet and GATA-3 in cells expressing either IFNg or IL-4/5/13 but not both. Naïve DO11.10 TCR-transgenic cells were sort-purified and activated with BMDCs and the cognate Ova-II peptide for 72 h without any ‘polarizing’ conditions. These primed cells were rested for 24 h in IL-2 (5 U/ml) before being tested for cytokine commitment.

**Fig 8:**
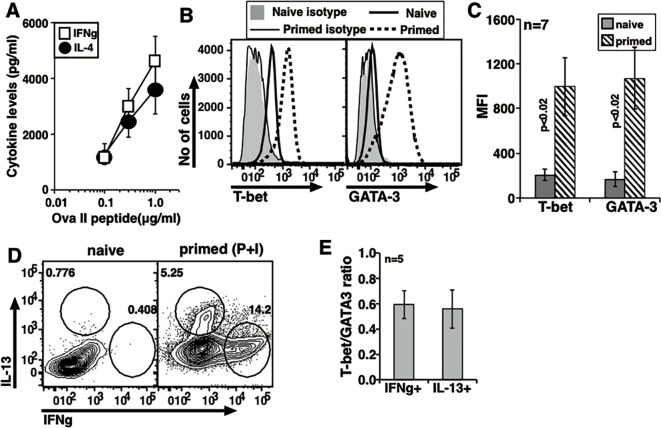
Peptide-MHC mediated non-polarizing activation mimics polyclonal activation pattern. (A) Dose response curve to show secreted cytokine levels in primed and recalled DO11.10 cells (mean ± s.e.). Data compiled from 5 independent experiments. (B) A representative profile to show T-bet and GATA-3 upregulation in naive and 72 h primed DO11.10 cells. (C) Data from independent experiments compiled to show T-bet and GATA-3 MFI in naive and primed DO11.10 cells. (D) Intracellular cytokine staining of naive and primed DO11.10 cells after P+I treatment. (E) Data from independent experiments compiled to show relative T-bet/GATA-3 ratios in IFNg+ and IL-13+ DO11.10 cells (mean ± s.e.).

When these in vitro-primed DO11.10 cells were restimulated with BMDCs and titrating concentrations of Ova-II peptide, they secreted substantial levels of both IFNg and IL-4 (Fig 8A). These cells showed unimodal distribution of T-bet and GATA-3 which was significantly higher than seen in DO11.10 naive cells (Fig 8B and 8C). T-bet and GATA-3 were co-expressed in these cells (S4C Fig). Primed cells differentiated well into IFNg-producing or IL-13-producing cell subsets (Fig 8D). However, in independent experiments performed the small population which appeared to co-express both IL-13 and IFNg was gated and the frequencies of single and dual cytokine positive cells were compared. The frequency of dual cytokine positive population was not different from that seen in naive DO11.10 cells whereas frequencies of IFNg+ and IL-13+ cells were significantly higher (S4D Fig). A representative overlay of IFNg+ and IL-13+ cells showing T-bet and GATA-3 staining indicates that despite being unimodal the transcription factor expression between the two cell populations is very similar (S4E Fig). Further, T-bet/GATA-3 ratios in IFNg-expressing and IL-13-expressing DO11.10 cells were not different from each other (Fig 8E).

We examined the issue further with mice carrying the OT-II TCR transgene bred into either the more ‘Th1’ B6 background (OTII.B6) or the more ‘Th2’ BALB.b background (OTII.BB) for 10 generations. Naive cells from OTII.B6 and OTII.BB mice were activated with Ova-II peptide and syngeneic BMDCs in vitro as above. The resultant primed OT-II cells from both backgrounds showed unimodal expression of T-bet and GATA-3 suggestive of co-expression in two color plots (S4F Fig) and the expression levels of T-bet and GATA-3 were significantly higher than those in the naïve cells (S4G Fig). Yet, these primed cells showed quite distinct IFNg/IL-4 balance commitment on restimulation as measured by ELISpot assays (S4H Fig) with no appreciably detectable dual cytokine-expressing cells.

### Longer exposure to or higher concentration of naive cell priming stimulus in vitro modifies cytokine balance commitment without radically altering TF expression patterns

**Fig 9:**
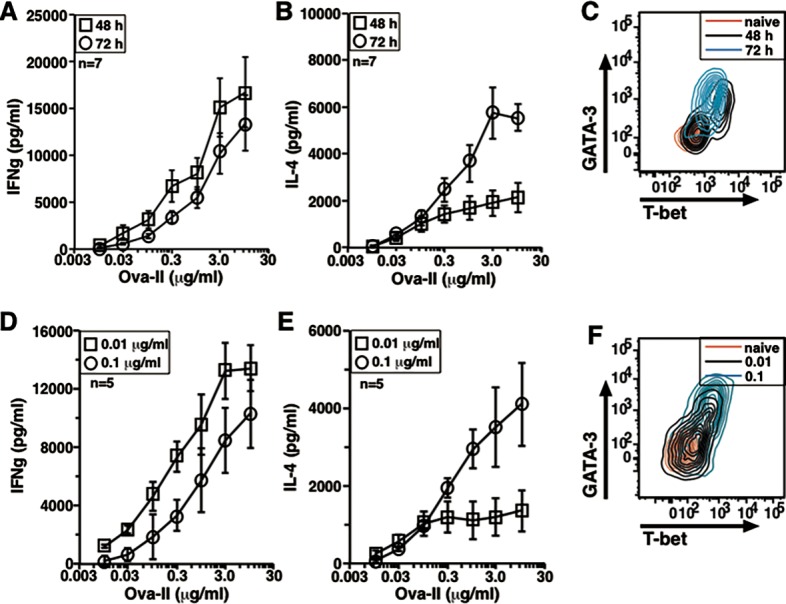
Variation in the duration and dose of antigenic exposure alters Th1/Th2 balance post-priming in vitro. (A) IFNg secretion pattern after 48 or 72 h activation of DO11.10 in vitro in recall response (mean ± s.e.). Data compiled from 7 experiments. (B) IL-4 secretion pattern after 48 or 72 h activation of DO11.10 in vitro during recall (mean ± s.e.). Data compiled from 7 experiments. (C) A representative two-color plot of T-bet and GATA-3 expression in naive, 48 h primed and 72 h primed DO11.10 cells as overlays. (D) IFNg secretion pattern after low or high dose of Ova-II mediated activation of DO11.10 in vitro during recall (mean ± s.e.). Data compiled from 5 experiments. (E) IL-4 secretion pattern after low or high dose of Ova-II mediated activation of DO11.10 in vitro during recall (mean ± s.e.). Data compiled from 5 experiments. (F) A representative two-color plot of T-bet and GATA-3 expression in DO11.10 cells primed with 0.01 and 0.1 μg/ml Ova-II peptide for 72 h along with naive cells as overlays.

Using the TCR-transgenic naive cell priming approach, we tested modifications of ligand concentration and exposure time that have been reported to alter the ‘Th1’/‘Th2’ cytokine commitment balance in responding cells [41–43]. Ova-II peptide was used to prime naïve DO11.10 cells in vitro for either 48 or 72 h before resting them in IL-2 for 24 h. Restimulation with cognate peptide and BMDCs for 24 h showed that longer duration of priming led to relative lowering of IFNg production and a clear increase in IL-4 production in a dose-dependent fashion indicative of a ‘Th2’ shift as compared to shorter priming period (Fig 9A and 9B). However, primed cells from both the groups showed substantially unimodal expression pattern for T-bet and GATA-3 and two color plots indicated similar co-expression of TFs in both the groups (Fig 9C).

Similarly, when naïve DO11.10 cells were primed with either a low (0.01 μg/ml) or a high (0.1 μg/ml) concentration of Ova-II peptide for 72 h, re-stimulation assays showed lower IFNg production and enhanced IL-4 production in a dose-dependent fashion suggestive of a ‘Th2’ shift in the cytokine commitment balance with the higher as compared to lower ligand concentration (Fig 9D and 9E). Yet, both cell populations showed unimodal increases in T-bet and GATA-3 with two color plots for TFs showing similar co-expression pattern in both the groups (Fig 9F). ELISpot assays also showed that higher peptide concentration led to higher number of IL-4 spots (S4I Fig) strengthening the observations made with secreted cytokines.

Together, these data demonstrated that memory cells generated in a wide variety of situations known to modulate the cytokine balance of their effector commitment showed unimodal co-expression of the major ‘Th1’/‘Th2’ program-regulating TFs T-bet and GATA-3, without necessarily co-expressing ‘Th1’ and ‘Th2’ cytokines.

## Discussion

CD4 T cells responses have been commonly categorized into different ‘subsets’ based on the cytokine groups they are committed to making on re-stimulation, such as ‘Th1’, ‘Th2’, ‘Th17’ or ‘Th9’. This categorization is supported by the fact that, by and large, primed ‘effector/memory’ CD4 T cells make one or the other of these cytokine groups, although exceptions are reported, such as ‘Th1+Th2’ [44] or ‘Th2+Th17’ [45]. Analyses of the molecular mechanisms responsible for these alternative commitments during differentiation have led to a model of differential induction of ‘master regulator’ TFs for each group, and the most studied example is of T-bet/Tbx21 for Th1 and GATA-3 for Th2 responses [46]. T-bet and GATA-3 are thought to function, in part, by repressing each other’s expression [47] thus driving differentiation.

If this model is correct for all situations, then memory CD4 cells should not, by and large, co-express T-bet and GATA-3. Our data here demonstrate that memory cells formed in vivo, both in mice and humans, show co-expression of T-bet and GATA-3 with each TF showing unimodal pattern. Even when these effector-memory cells express either IFNg or IL-4/5/13 but not both, they co-express these TFs. Analysis of antigen-specific CD4 T cells generated in vivo following immunization also shows the same pattern. GATA-3 co-expression in human Th1 cells expressing T-bet has been documented [48] and one possible molecular explanation is that the binding sites of GATA-3 in Th1 and Th2 cells are different [49]. Our data suggest that naturally primed mouse memory CD4 cells may also behave similarly. Further, our data show that when polyclonal naive cells are primed in vitro under non-polarizing conditions, they generate effector cells that resemble memory cells generated in vivo. Amounts of cytokines secreted by in vivo generated memory cells are comparable to in vitro primed cells and much lower than in vitro polarized cells. Effector memory cells generated in vitro in non-polarizing conditions also show unimodal upregulation and co-expression of T-bet and GATA-3 to a similar extent in cells producing either IFNg or IL-4/5/13. This is not the case only with the artificial priming conditions using plate-bound antibodies, but also in the more physiological situation of DC- and cognate peptide-mediated priming of TCR-transgenic naïve CD4 cells. Such memory cells generated in vitro using a variety of ‘non-polarizing’ or ‘neutral’ activation protocols [47,50–55] are commonly referred to as ‘Th0’ memory cells. Unlike these cells, memory cells formed under polarizing conditions do indeed show, in addition to mutually exclusive expression of IFNg versus IL-4/IL-13, mutually exclusive expression of T-bet and GATA-3 as well. However, our data, including those on antigen-specific cells generated in vivo, indicate the possibility that most common outcomes of naïve CD4 cell priming in vivo may be the generation of these ‘Th0’-like memory cells, and that mutually exclusive expression of T-bet and GATA-3 is unlikely to be the major basis of their generation. Co-expression of T-bet and GATA-3 in association with co-expression of ‘Th1’ and ‘Th2’ cytokines has been reported earlier [23,56–58], unlike the present data. While both IFNg-expressing and IL-13-expressing memory CD4 T cells in mice and humans co-express T-bet and GATA-3, there are quantitative differences in the relative levels of the two transcription factors. Our data suggest that the quantitative T-bet/GATA-3 balances are altered between IFNg-expressing versus IL-13-expressing memory CD4 T cells, rather than mutually exclusive expression of T-bet and GATA-3. These data raise the question of how quantitative modulation of low levels of T-bet and GATA-3 in the same cell influence the cellular programming so as to result in re-stimulation-driven expression of either ‘Th1’ cytokines or ‘Th2’ cytokines but not both.

A substantial frequency of the so-called ‘Th0’ memory cells co-expressing T-bet and GATA-3 do not express either IFNg or IL-4,5,-13 in our hands, a situation that has also been reported in antigen-specific memory cell recall situations in vitro and in vivo [23,59]. The prominence of these ‘Th0’ memory cells also invites speculations about the possible role of such Th0 commitment in the many examples of memory cell cytokine program plasticity that have been reported [1,19–22].

It must be noted that a number of infections/immunization protocols in mouse models report generation of antigen-specific polarized ‘Th1’ or ‘Th2’ memory cells [58–60]. It is therefore probable that a number of alternative pathways are involved in regulating these commitment programs. In this context it is interesting that, for a number of genetic and other situations reported to alter the ‘Th1’/‘Th2’ cytokine balance, such as the well-known differences between the B6 versus BALB or FVB genetic backgrounds or between young versus aged animals, not too many underlying molecular mechanisms have been elucidated. Some of these mechanisms are suggested to be due to CD4 T cell-extrinsic APC-microenvironmental distinctions [1–3,10,13], and the few CD4 T cell-intrinsic differences reported do not necessarily involve T-bet- and/or GATA-3-based distinctions [15]. On this background, it is noteworthy that our data show that such B6/BALB/FVB or young/aged differences in the balance of ‘Th1’/‘Th2’ cytokine commitment are detectable in APC-free priming with significantly higher levels of GATA-3 expression in Th2 committed cells as a common phenomenon.

Our data also show interesting nuances in the role of APC-derived factors. Clearly, ligand dose/time-based differences, reported to be able to affect ‘Th1’/‘Th2’ cytokine balances in responding CD4 T cells [39–41], perform similar roles in our data despite T-bet/GATA-3 co-expression. Our data with IFNg-null mice show that the role of IFNg in naïve CD4 cell priming is likely to be extrinsic to naive cells, since memory cells from these mice show higher levels of ‘Th2’ cytokines, but memory cells generated in vitro do not. Again, both memory cell populations from IFNg-null mice show co-expression of T-bet and GATA-3, similar to B6 mice.

While transcription of T-bet and GATA-3 on their own are sufficient for expression of Th1 and Th2 cytokines respectively [61], the data presented here clearly show that it is not necessary for them to negatively regulate each other’s expression for appropriate functioning of the differentiated cells.

Co-expression of T-bet and GATA-3 has been demonstrated previously [23], and that all effector CD4 T cells, even in polarized situations, do not necessarily express their respective cytokines [44,62,63]. Peine et al. [23] demonstrate situations in which co-expression of T-bet and GATA-3 leads to co-expression of IFNg and IL-4/5/13 from individual effector-memory CD4 T cells, whereas Hegazy et al. [44] show that GATA-3-expressing ‘Th2’ cells can be reprogrammed in vivo to co-express T-bet and to express both ‘Th1’ and ‘Th2’ cytokines. Lai et al. [62] show that T-bet activity is not essential for early IFNg expression from committed effector-memory CD4 T cells, and Zhu et al. [63] show that all effector CD4 T cells, even in polarized situations, do not necessarily express their respective cytokines. The novelty in our findings lies in the fact that we show that in response to many situations of immune priming both in vivo and in vitro, even when the resultant effector/memory CD4 T cells make either IFNg or IL-4/5/13, they show co-expression of both T-bet and GATA-3 at comparable levels.

In conclusion, our data show that low-level co-expression of the TFs T-bet and GATA-3 is commonly to be found in memory CD4 cells generated both in vivo and in vitro. Yet, such co-expression does not induce co-expression of the cytokines that these TFs regulate. These data indicate that models of mutually exclusive expression and function of T-bet or GATA-3 are likely to be insufficient to explain natural CD4 T cell priming outcomes.

## Supporting information

S1 Fig Memory cells from B6, B6-aged and FVB mice show co-expression of TFs but no co-expression of cytokines. (A) Representative staining for sorted naive and memory CD4 T cells from B6 mice to show isotype controls for GATA-3, T-bet, IFNg and IL-4,5,13 staining. Numbers in the brackets indicate MFI values. (B) Overlays for naive and memory CD4 T cells from B6 mice showing T-bet and GATA-3 staining pattern. Data representative of many independent experiments. (C) Overlay of profiles of naive, IFNg+ memory and IL-4,5,13+ve memory CD4 T cells from B6 mice to show T-bet and GATA-3 staining pattern following P+I stimulation. Data representative of many independent experiments. (D) Dual colour ELISpot data from one experiment showing number of spots (mean ± s.e.) for IFNg and IL-4 from in vivo generated memory cells for different strains of mice as indicated. Pattern representative of 2 independent experiments. No dual positive spots were detectable. (E) Overlays for naive and in vivo generated memory CD4 T cells from different strains showing T-bet and GATA-3 staining pattern. Data representative of 6 independent experiments.
(TIF)

S2 Fig Wild type, 4Get and IFNg-null mice do not co-express cytokines but co-express TFs. (A) A representative staining profile of CD4+CD44+ cells from BALB/c and 4Get mice to show GFP expression. (B) A representative profile of GFP+ve and GFP-ve cells as overlays. Cells were sorted from CD4+CD44+ cells from 4Get mice. (C) Intracellular staining for IFNg and IL-4,5,13 in sorted GFP-ve and GFP+ve populations, without P+I treatment. Data representative of 6 experiments. (D) Data from one representative experiment out of 3 to show secreted cytokines from sorted MCD4T cells from B6 and IFNg-null mice. A dose response shown for anti-CD3 stimulation as described in Materials and methods. (E) Overlays for naive and in vivo generated memory CD4 T cells showing T-bet and GATA-3 staining pattern. Data representative of three independent experiments.
(TIF)

S3 Fig In vitro generated effector cells express higher levels of TFs as compared to naive cells and IL-2 does not change the outcome. (A-D) Representative staining for human naive and ex vivo memory CD4 T cells to show isotype controls for T-bet, GATA-3, IFNg and IL-4,5,13 staining. Numbers in the brackets indicate MFI values. (E) CFSE dilution profile of in vitro activated naive CD4 cells from B6 mice at the end of 72 h. (F) CD44 upregulation on CFSE diluted cells from (E). Profiles in (E) and (F) representative of many experiments. (G-H) MFI values for T-bet and GATA-3 for BALB/c (G) or DO11.10 (H) NCD4 T cells primed in vitro, with anti-CD3 and anti-CD28 or cognate peptide & DCs respectively, in presence or absence of IL-2 as shown. (mean ± s.e., n = 3, n.s., not significant). (I-J) Representative staining for naive and in vitro primed CD4 T cells from B6 mice to show isotype controls for T-bet and GATA-3. Numbers in the brackets indicate MFI values. (K) A representative two-colour plot of T-bet and GATA-3 expression in naive and primed cells from B6 mice as overlays. (L-M) Representative staining for naive and in vitro primed CD4 T cells from B6 mice to show isotype controls for IFNg and IL-4,5,13. Numbers in the brackets indicate MFI values.
(TIF)

S4 Fig Cytokines are not co-expressed by TFs are in NCD4 T cells from many strains primed in vitro. (A) Superimposed histograms of IFNg+ve and IL-13+ve cells from polarised and non-polarised cells to show apparent differences in IFNg MFIs but no reproducible differences in IL-13 MFIs. Data representative of 2 experiments of polarised and non-polarised activation done in parallel. (B) Dual colour ELISpot data from one experiment showing number of spots (mean ± s.e.) for IFNg and IL-4 from in vitro primed CD4 T cells for different strains of mice as indicated. Pattern representative of 2 independent experiments. No dual positive spots were detectable. (C) A representative two-colour plot of T-bet and GATA-3 expression in naive and in vitro primed DO11.10 cells as overlays. Data representative of 6 experiments. (D) Frequencies of IFNg+, IL-13+ or IFNg & IL-13 dual positive DO11.10 cells from unstimulated (naive) or peptide+DC stimulated (primed) cultures. (mean ± s.e., n = 3, p values as shown) (E) A representative two-colour plot of T-bet and GATA-3 expression in IFNg+ and IL-13+ expressing in vitro primed DO11.10 cells following P+I treatment as overlays. Data representative of 5 experiments. (F) A representative two-colour plot of T-bet and GATA-3 expression in naive and primed OTII.B6 and OTII.BB cells as overlays. (G) Data from naive and in vitro activated OT-II.B6 and OT-II.BB cells (memory) to show T-bet and GATA-3 MFI values (mean ± s.e.). Data representative of 3–4 independent experiments. Isotype control values for naive and in vitro primed OT-II.B6 and OT-II.BB cells were comparable. (H) Dual colour ELISpot data from one experiment showing number of spots (mean ± s.e.) for IFNg and IL-4 from in vitro primed OT-II T cells from B6 and BALB.b backgrounds. Pattern representative of 2 independent experiments. No dual positive spots were detectable. (I) Dual colour ELISpot data from one experiment showing lower no. of spots for IFNg and higher no. of spots for IL-4 at higher dose (0.1 μg/ml) as compared to lower dose. No dual positive spots were detectable.
(TIF)
