# Supplementary figures and images for: Effector/memory CD4 T cells making either Th1 or Th2 cytokines commonly co-express T-bet and GATA-3
Source: PLoS One. 2017 Oct 31;12(10):e0185932. doi: 10.1371/journal.pone.0185932 (PMC5663332; doi:10.1371/journal.pone.0185932)

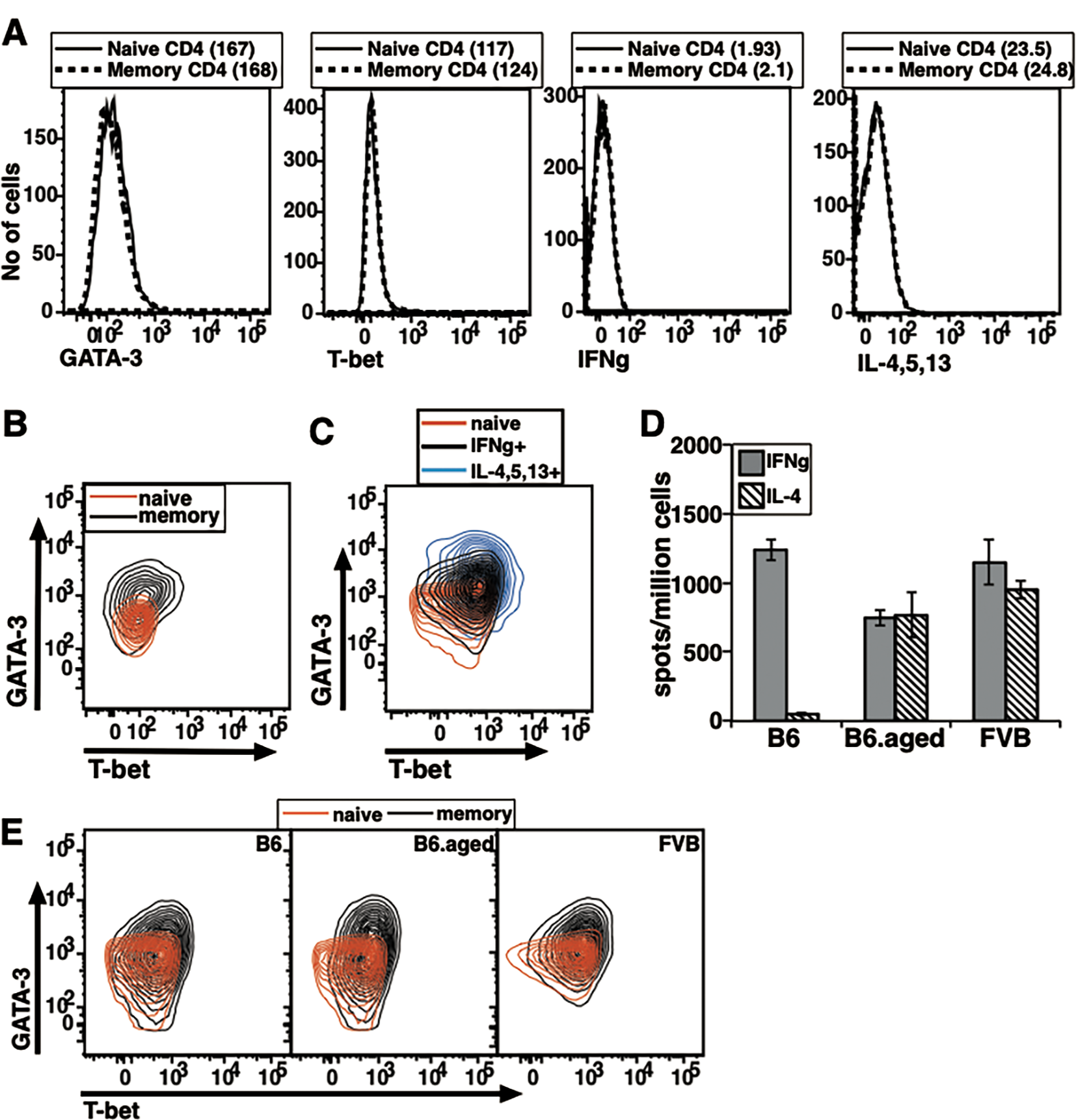

Supplement: S1 Fig — (A) Representative staining for sorted naive and memory CD4 T cells from B6 mice to show isotype controls for GATA-3, T-bet, IFNg and IL-4,5,13 staining. Numbers in the brackets indicate MFI values. (B) Overlays for naive and memory CD4 T cells from B6 mice showing T-bet and GATA-3 staining pattern. Data representative of many independent experiments. (C) Overlay of profiles of naive, IFNg+ memory and IL-4,5,13+ve memory CD4 T cells from B6 mice to show T-bet and GATA-3 staining pattern following P+I stimulation. Data representative of many independent experiments. (D) Dual colour ELISpot data from one experiment showing number of spots (mean ± s.e.) for IFNg and IL-4 from in vivo generated memory cells for different strains of mice as indicated. Pattern representative of 2 independent experiments. No dual positive spots were detectable. (E) Overlays for naive and in vivo generated memory CD4 T cells from different strains showing T-bet and GATA-3 staining pattern. Data representative of 6 independent experiments. (TIF) [file pone.0185932.s001.tif]

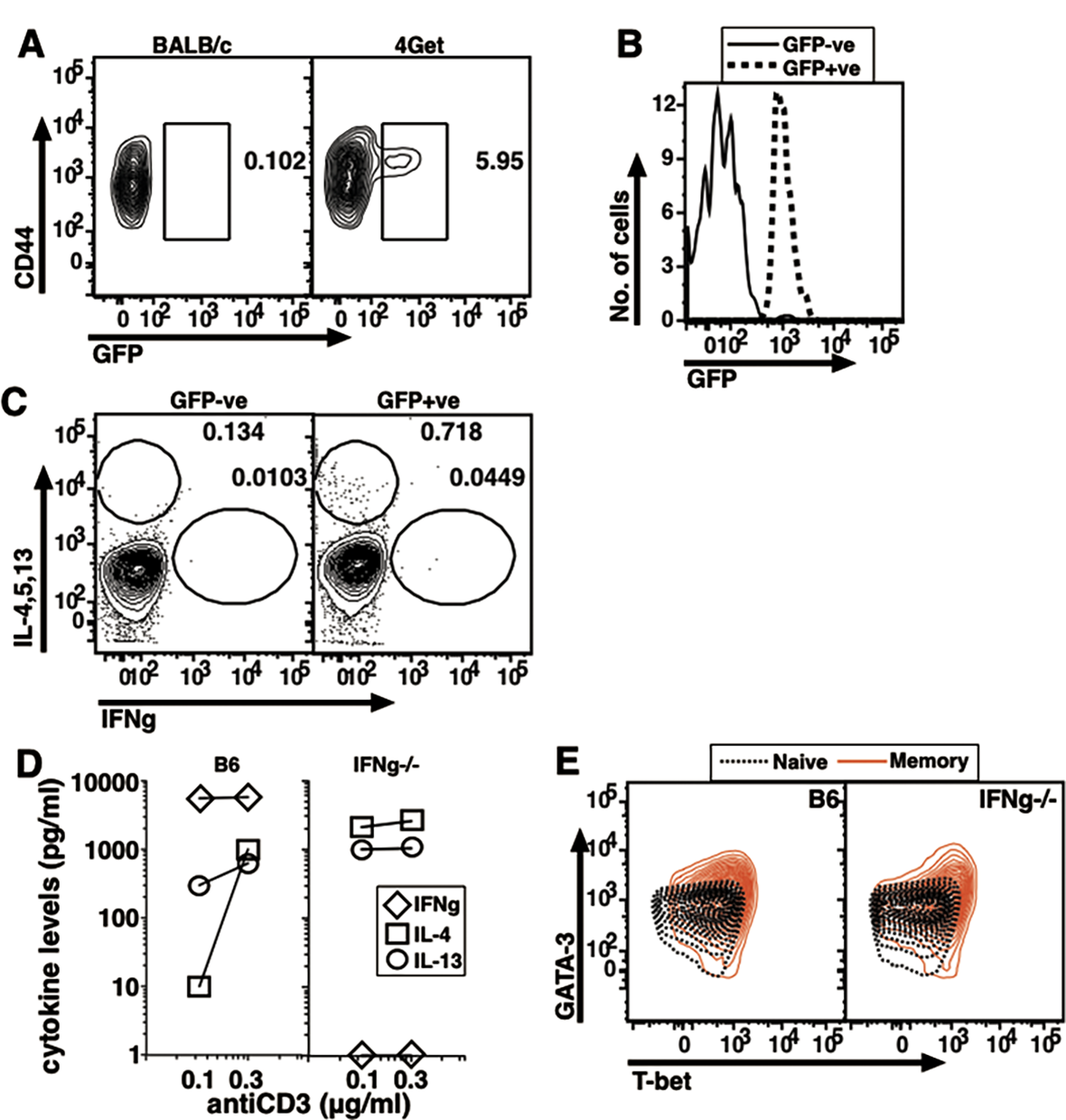

Supplement: S2 Fig — (A) A representative staining profile of CD4+CD44+ cells from BALB/c and 4Get mice to show GFP expression. (B) A representative profile of GFP+ve and GFP-ve cells as overlays. Cells were sorted from CD4+CD44+ cells from 4Get mice. (C) Intracellular staining for IFNg and IL-4,5,13 in sorted GFP-ve and GFP+ve populations, without P+I treatment. Data representative of 6 experiments. (D) Data from one representative experiment out of 3 to show secreted cytokines from sorted MCD4T cells from B6 and IFNg-null mice. A dose response shown for anti-CD3 stimulation as described in Materials and methods. (E) Overlays for naive and in vivo generated memory CD4 T cells showing T-bet and GATA-3 staining pattern. Data representative of three independent experiments. (TIF) [file pone.0185932.s002.tif]

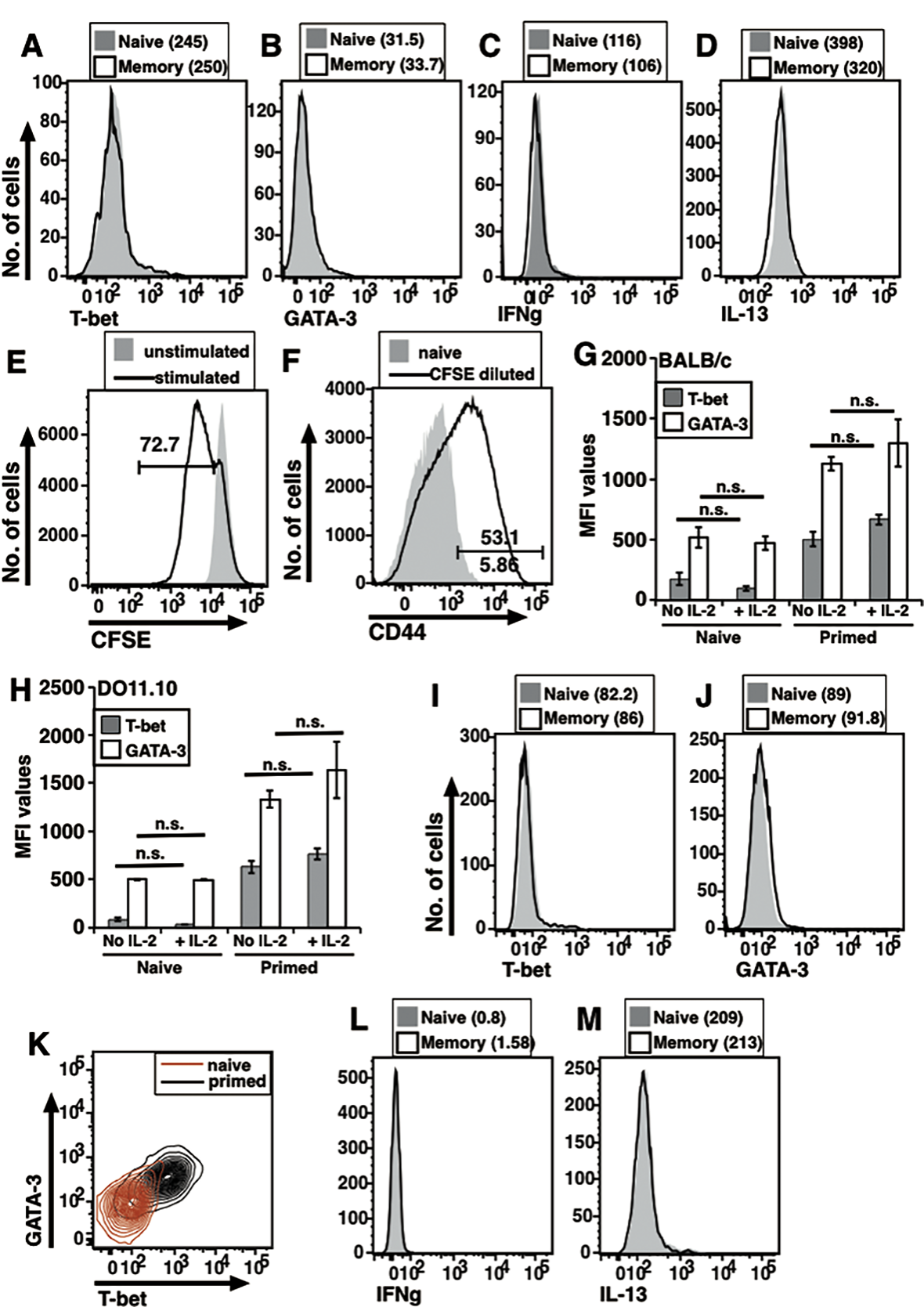

Supplement: S3 Fig — (A-D) Representative staining for human naive and ex vivo memory CD4 T cells to show isotype controls for T-bet, GATA-3, IFNg and IL-4,5,13 staining. Numbers in the brackets indicate MFI values. (E) CFSE dilution profile of in vitro activated naive CD4 cells from B6 mice at the end of 72 h. (F) CD44 upregulation on CFSE diluted cells from (E). Profiles in (E) and (F) representative of many experiments. (G-H) MFI values for T-bet and GATA-3 for BALB/c (G) or DO11.10 (H) NCD4 T cells primed in vitro, with anti-CD3 and anti-CD28 or cognate peptide & DCs respectively, in presence or absence of IL-2 as shown. (mean ± s.e., n = 3, n.s., not significant). (I-J) Representative staining for naive and in vitro primed CD4 T cells from B6 mice to show isotype controls for T-bet and GATA-3. Numbers in the brackets indicate MFI values. (K) A representative two-colour plot of T-bet and GATA-3 expression in naive and primed cells from B6 mice as overlays. (L-M) Representative staining for naive and in vitro primed CD4 T cells from B6 mice to show isotype controls for IFNg and IL-4,5,13. Numbers in the brackets indicate MFI values. (TIF) [file pone.0185932.s003.tif]

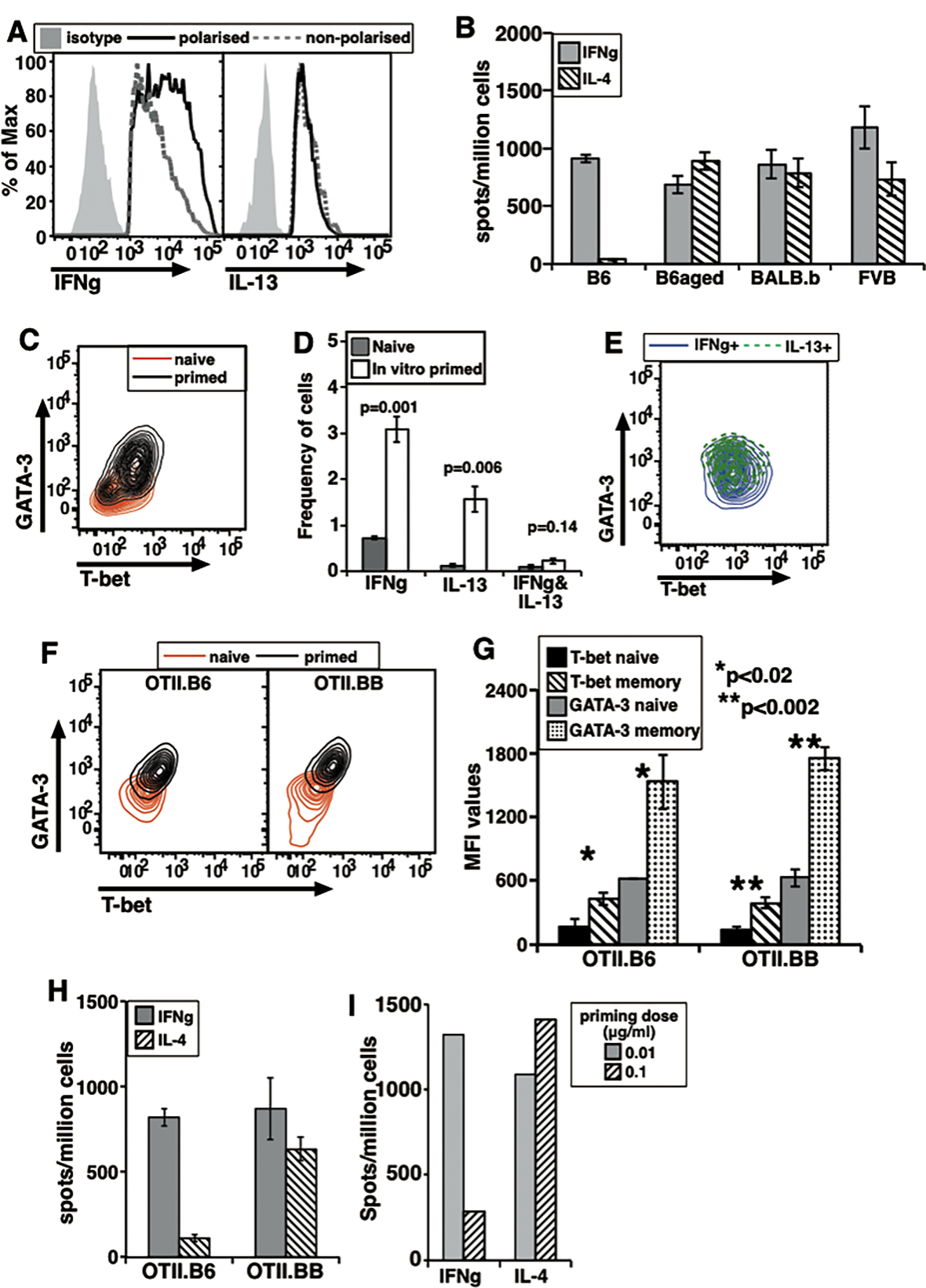

Supplement: S4 Fig — (A) Superimposed histograms of IFNg+ve and IL-13+ve cells from polarised and non-polarised cells to show apparent differences in IFNg MFIs but no reproducible differences in IL-13 MFIs. Data representative of 2 experiments of polarised and non-polarised activation done in parallel. (B) Dual colour ELISpot data from one experiment showing number of spots (mean ± s.e.) for IFNg and IL-4 from in vitro primed CD4 T cells for different strains of mice as indicated. Pattern representative of 2 independent experiments. No dual positive spots were detectable. (C) A representative two-colour plot of T-bet and GATA-3 expression in naive and in vitro primed DO11.10 cells as overlays. Data representative of 6 experiments. (D) Frequencies of IFNg+, IL-13+ or IFNg & IL-13 dual positive DO11.10 cells from unstimulated (naive) or peptide+DC stimulated (primed) cultures. (mean ± s.e., n = 3, p values as shown) (E) A representative two-colour plot of T-bet and GATA-3 expression in IFNg+ and IL-13+ expressing in vitro primed DO11.10 cells following P+I treatment as overlays. Data representative of 5 experiments. (F) A representative two-colour plot of T-bet and GATA-3 expression in naive and primed OTII.B6 and OTII.BB cells as overlays. (G) Data from naive and in vitro activated OT-II.B6 and OT-II.BB cells (memory) to show T-bet and GATA-3 MFI values (mean ± s.e.). Data representative of 3–4 independent experiments. Isotype control values for naive and in vitro primed OT-II.B6 and OT-II.BB cells were comparable. (H) Dual colour ELISpot data from one experiment showing number of spots (mean ± s.e.) for IFNg and IL-4 from in vitro primed OT-II T cells from B6 and BALB.b backgrounds. Pattern representative of 2 independent experiments. No dual positive spots were detectable. (I) Dual colour ELISpot data from one experiment showing lower no. of spots for IFNg and higher no. of spots for IL-4 at higher dose (0.1 μg/ml) as compared to lower dose. No dual positive spots were detectable. (TIF [file pone.0185932.s004.tif]
